# Solitary pleural tuberculoma diagnosed by thoracoscopic surgical resection

**DOI:** 10.1093/jscr/rjab408

**Published:** 2021-09-22

**Authors:** Ryusuke Sumiya, Satoshi Nagasaka, Takeshi Ikeda, Hideki Miyazaki

**Affiliations:** Department of General Thoracic Surgery, National Center for Global Health and Medicine, Tokyo, Japan; Department of General Thoracic Surgery, National Center for Global Health and Medicine, Tokyo, Japan; Department of General Thoracic Surgery, National Center for Global Health and Medicine, Tokyo, Japan; Pathology Division of Clinical Laboratory, National Center for Global Health and Medicine, Tokyo, Japan

## Abstract

Tuberculoma is a manifestation of pleural tuberculosis. Although the clinical manifestation of tuberculoma has been widely reported, the pathogenesis of this condition still remains unclear. An abnormal shadow was detected on the chest radiograph of a 44-year-old man with a history of pulmonary tuberculosis. Computed tomography revealed a well-defined, elliptical 44 mm nodule located in the right posterior thoracic cavity. Thoracoscopic surgery was performed to rule out malignant tumors. Although loose adhesions were observed throughout the thoracic cavity, a nodule was found between the visceral pleura and parietal pleura. En bloc resection was performed, and the patient was pathologically diagnosed with tuberculoma. An acid-fast bacterium culture was negative, and the patient’s recovery was uneventful without chemotherapy. Surgical resection should be considered to rule out malignancy, because tuberculomas are difficult to distinguish from malignant pleural tumors.

## INTRODUCTION

Tuberculosis is a common infection, not only in developing countries, but also in a part of developed countries. Although the major manifestation of tuberculosis is pulmonary tuberculosis, extrapulmonary tuberculosis, such as pleural and lymph node tuberculosis, is not rare, accounting for 25% of the total [1]. Pleural tuberculosis as primary and post-primary tuberculosis showed pleural effusion and thickening on radiological imaging. In addition, some cases of pleural tuberculosis have nodular lesions in the pleura, and can be called pleural tuberculoma. Although the clinical manifestation of tuberculoma has been reported so far, the pathogenesis of this condition remains unclear [2]. Here, we present a rare case of surgically resected pleural tuberculoma with operative and pathological findings.

## CASE PRESENTATION

**Figure 1:**
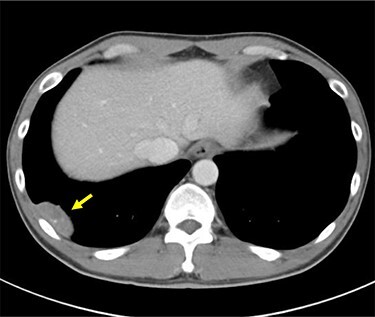
Preoperative enhanced CT scan demonstrates a well-defined 44 mm solid nodule with calcification in the right lateral thoracic cavity (yellow arrow). CT: computed tomography.

**Figure 2:**
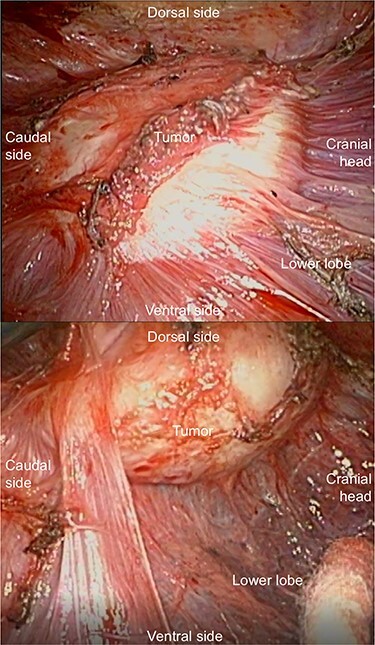
The nodule is observed between the visceral pleura and parietal pleura.

**Figure 3:**
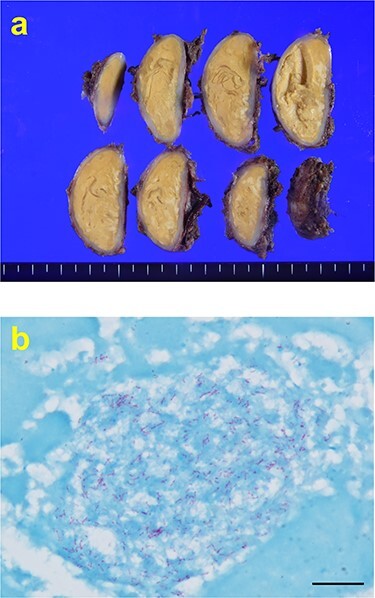
(**a**) Macroscopically, encapsulated chronic granulomatous with caseous necrosis. (**b**) Ziehl–Neelsen staining (1000× magnification) showing red bacillus. Scale bar = 10 μm.

An abnormal shadow was detected on the chest radiograph of a 44-year-old man with a history of pulmonary tuberculosis, who underwent a whole-body chemotherapy regimen of rifampicin, isoniazid, ethambutol and pyrazinamide at age 42. He was a former smoker with 10 pack-years. Physical examination and routine laboratory tests did not reveal any abnormalities. Both chest radiographs during chemotherapy and completion of treatment showed consolidation in the right upper field and a 44 mm nodule in the right lower lung field with no interval change. Enhanced computed tomography (CT) revealed a well-defined elliptical nodule (44 × 20 mm) located in the right posterior thoracic cavity (Fig. 1). A CT scan also demonstrated bilateral consolidation in the upper lobe. Pleural tuberculoma, solitary fibrous tumor or malignant tumor was suspected, and thoracoscopic resection of the chest wall tumor was performed. Intraoperatively, a nodule was found between the visceral pleura and parietal pleura, although loose adhesion was observed throughout the entire thoracic cavity (Fig. 2). There was no disseminated disease in the thoracic cavity. It was determined to be noninvasive, and en bloc resection was performed. Pathological findings showed capsuled chronic granulomatous inflammation with Langhans giant cells and caseous necrosis (Fig. 3a). Ziehl–Neelsen staining revealed a red bacillus, resulting in pleural tuberculoma diagnosis (Fig. 3b). Smears for acid-fast bacilli and PCR for tuberculosis were positive. However, an acid-fast bacterial culture was negative within 6 weeks, and the patient’s recovery was uneventful after a 2-month follow-up period without chemotherapy.

## DISCUSSION

Pulmonary tuberculosis is a common infection, and pleural tuberculosis is a common extrapulmonary manifestation [2]. Pleural tuberculoma is described as pleural tuberculosis-related pleural nodular lesions, which is observed in >10% cases. Pleural tuberculoma often occurs within 3 months after the initiation of antituberculosis chemotherapy, and the tumor typically improves within 6 months to 1 year without medical intervention. However, as only a few surgical and pathological findings have been reported regarding tuberculoma, little is known about them [3].

Some reports mentioned that the basis of surgical resection is a reduction in infectiousness [4]. However, there have been few reports of tuberculoma in the recent past, and little is known about the pathogenesis of tuberculoma. A recent report revealed that *Mycobacterium tuberculosis* could be isolated from resected tuberculomas in 85% of cases [4]. In contrast, there has been no report that the case had a positive culture of *M. tuberculosis*, and several reports in Japanese literature revealed that the culture of acid-fast bacilli is typically negative in tuberculoma [3, 5]. Almost all cases of pleural tuberculomas arise during or after the course of antituberculous therapy [3], and several cases have been reported without chemotherapy [2]. Based on these reports, the opinion that hypersensitivity reaction to the dead cells of *M. tuberculosis* being the cause of pleural tuberculomas is considered to be the most widely accepted theory. Resolution of pleural tuberculomas without medical intervention was observed in almost all cases. Therefore, surgical resection has been used for diagnostic purposes in the recent years.

Several cases of tuberculoma were reported based on radiological findings, which described tuberculomas as masses on the pleura with or without calcification [2]. Therefore, it is important to consider the possibility of a pleural tumor as a differential diagnosis, such as solitary fibrous tumor and malignant pleural mesothelioma [6, 7]. In some cases, it is difficult to distinguish it from intra-lung tumors because the mass often juts out into the lung. Furthermore, a previous report revealed that patients with pulmonary tuberculosis have a higher risk of lung cancer [8]. Therefore, a diagnosis of tuberculoma should be made cautiously. Clinically, however, it is difficult to diagnose pleural tuberculoma in some cases; thus, needle biopsy or surgical resection should be considered if atypical imaging or course is observed. In the present case, tuberculoma was difficult to diagnose with clinical findings because the nodule on the pleura with calcification did not decrease in size more than a year after chemotherapy, and hence, surgical resection was able to distinguish it from a pleural malignant tumor.

As the previous Japanese report mentioned, the surgical and pathological findings in this report confirmed thickening of the pleura or connection from the pleura was not observed in tuberculomas, although it had adhesion with the visceral pleura and parietal pleura [3]. This feature helped in en bloc resection and distinguishing it from empyema and malignant tumor intraoperatively. Furthermore, the small tumor size and peripheral localization enabled us to resect the tumor with minimally invasive endoscopic surgery.

In conclusion, tuberculosis is still considered a leading cause of death worldwide. We should consider pleural tuberculoma as a differential diagnosis. If it is difficult to distinguish pleural tuberculoma from malignant pleural tumors, surgical resection as biopsy should be considered to rule out malignancy. Endoscopic surgery has potential as a suitable treatment approach to resect tuberculomas.
